# Epidemiology of cervical cancer in Iran in 2016: A nationwide study of incidence and regional variation

**DOI:** 10.1002/cnr2.1973

**Published:** 2024-01-12

**Authors:** Alireza Anjam Majoumerd, Saeed Hosseini, Seyyed Mohammad Hossein Hosseini, Gholamreza Roshandel, Vahid Rahmanian, Narjes Hazar

**Affiliations:** ^1^ School of Medicine Shahid Sadoughi University of Medical Sciences Yazd Iran; ^2^ Center for Healthcare Data Modeling, Department of Biostatistics and Epidemiology, School of Public Health Shahid Sadoughi University of Medical Sciences Yazd Iran; ^3^ Department of Epidemiology, School of Public Health Iran University of Medical Sciences Tehran Iran; ^4^ Department of Biostatistics and Epidemiology Shahid Sadoughi University of Medical Sciences Yazd Iran; ^5^ Golestan Research Center of Gastroenterology and Hepatology Golestan University of Medical Sciences Gorgan Iran; ^6^ Department of Public Health Torbat Jam Faculty of Medical Sciences Torbat Jam Iran; ^7^ Diabetes Research Center Shahid Sadoughi University of Medical Sciences Yazd Iran

**Keywords:** cervical cancer, epidemiology, incidence, Iran

## Abstract

**Background:**

Cervical cancer is a common, and fatal cancer in women worldwide. This cancer is the fourth most common cancer in women after breast, colorectal, and lung cancer.

**Aims:**

This study aims to investigate the age‐standardized incidence rate (ASR) and the geographical distribution of Cervical Cancer in Iran.

**Methods:**

This study was designed as a descriptive cross‐sectional investigation. The study sample comprised all individuals registered as cervical cancer patients in the National Cancer Registry system in 2016. The crude rate and ASR for each province were computed independently. Furthermore, we employed ArcMap10.5 software and geographic information system to conduct an analysis of the gathered data. In order to ascertain the spatial distribution and clustering of cervical cancer incidence, we utilized Moran's *I*, which measures spatial autocorrelation.

**Results:**

We studied a total of 808 cases of cervical cancer with a median age to be 52.19 years (IQR≈1.35). Among these cases, 685 (84.7%) were diagnosed based on the pathological reports with morphological verification, while 81 patients (10.1%) were clinically identified, and 42 cases (5.2%) were diagnosed using the death certificate‐only method. Squamous cell carcinoma accounted for 61% of all cases (*n* = 497). The ASR of cervical cancer in Iran was 1.90 per 100 000 populations. The provincial ASR ranged from 0.29 to 5.03 per 100 000, with the highest rates observed in Golestan (5.03), East Azerbaijan (4.07), and Ilam (3.72). We found no clustering patterns in the distribution of provincial crude, age‐specific, and age‐standardized incidence rates (*p* > .05).

**Conclusion:**

The incidence of cervical cancer in Iran was lower than the global average, and we did not identify any significant disparities in the incidence rates among the provinces. Although there were differences in incidence rates among the areas, these were not clustered. It is crucial to remember that cervical cancer is still a major public health issue in Iran, and in order to lessen the disease's burden, national initiatives to enhance screening, early identification, and access to efficient treatment should continue to be top priorities.

## 1 INTRODUCTION

Cervical cancer is a common and fatal cancer in women worldwide.^1^ This cancer is the fourth most common cancer in women (after breast cancer [2.1 million cases], colorectal cancer [0.8 million], and lung cancer [0.7 million]). About 570 000 cases of cervical cancer and 311 000 deaths from the disease occurred worldwide in 2018.^2^ ASR of this cancer is 13.1 per 100 000 people worldwide, and this rate widely varies among different countries (2–75 cases per 100 000 women).^2^

Based on the literature, most cervical cancer cases, and the highest mortality related to this disease occur in low‐ and middle‐income countries.^3^ About 90% of deaths from this cancer occurs in developing countries, and 144 400 deaths occur in Asia.^4^ Cervical cancer causes 2.4 million years of life loss among women 25–64 years old.^3^ The average age of patients with this cancer is 52.2 years, and the peak incidence in Iran is between 50 and 55 years.^5^ Invasive cervical cancer is preventable in terms of its long period before the invasion, preventable risk factors, efficiency of screening programs, and appropriate treatment of primary lesions.^6^

Regarding the Iranian Cancer Society report in 2006, the prevalence of cervical cancer was 6–7 out of every 100 000 cases.^3^ The incidence of cervical cancer among younger women has increased in recent years in Iran.^3^ The incidence of cervical cancer in Iran is not high compared with other countries, which is not among the 12 most common cancers in the country.^2^, ^7^ Nevertheless, the need to tackle this disease in our nation has arisen as a result of the potential for primary prevention (such as self‐control and vaccine injection) on one hand, and the option of early screening and treatment on the other hand.^1^ The usefulness of preventive measures was proven in Western countries. One of the best examples is NHS England, which first targeted to diminish the disease burden via a national cervical cancer screening program in 1988, leading to a prominent reduction in more than a third of cases in England. This program is now available for 25 and over year older women, as cervical cancer is rare at younger ages.^8^

World Health Organization reports that most cervical cancer cases, precisely 85%, are found in developing nations. Significant disparities in this ailment's occurrence and fatality rates exist between developed and developing countries.^7^ While foreign studies could not shed light on the disease situation in Iran,^5^ it is evident that cancer prevalence is on the rise in the country. However, there has been a remarkable lack of research that have investigated the trajectory of this serious illness and changes in its prevalence and epidemiological features in recent decades. Furthermore, there is no current data on the incidence of this cancer in the country by province.^9^ The majority of present research was limited to specific regions, which has involved relatively small sample sizes. Comprehensive epidemiological data are imperative for effective planning and strategic decision‐making. Therefore, this study aimed to determine ASR and the geographical distribution of cervical cancer in Iran.

## 2 MATERIALS AND METHODS

### 2.1 Study design

This study was a descriptive cross‐sectional investigation encompassing all instances of invasive cervical cancer recorded within the Iranian National Population‐based Cancer Registry (INPCR) for 2016. The data processing for this specific year was conducted over a period spanning from 2018 to 2020. Following that, in 2021, the Annual Report of the INPCR for 2016 was officially released. The data gathering process included obtaining the required permissions and completing administrative communication in order to access relevant information inside the INPCR database. This information encompassed key variables, such as age, morphology code, method of diagnosis, and the province of residence, for individuals diagnosed with cervical cancer. The methods of data collection in the INPCR were previously described.^10^

### 2.2 Definition

It states that the classification of cervical cancer was based on International Statistical Classification of Diseases and Related Health Problems 10th Revision (ICD‐10), specifically the code C53. This code is used to classify malignant neoplasms of the cervix.^11^

### 2.3 Crude incidence and age‐specific incidence rate

The crude incidence rate is the number of new cases of a disease (in this case, cervical cancer) that occur in a population over a specific period, typically expressed as a rate per 100 000 people. To calculate the crude incidence rate of cervical cancer in each province, we divided the number of new cases of cervical cancer in that province by the population of that province, and then multiplied the result by 100 000.

The age‐specific incidence rate (ASIR) is the number of new cases of a disease that occur in a specific age group within a population over a particular period, typically expressed as a rate per 100 000 people in that age group.^12^ We divided the number of new instances of cervical cancer in each age group by the number of people in that age group in the province, and then multiplied the result by 100 000 to get the age‐specific incidence rate of cervical cancer for each province. ASIR is a summary measure of the incidence rate that considers the age structure of the population.^13^ ASIR allows for the comparisons of disease rates among the populations with different age distributions. To calculate the provincial ASIRs for cervical cancer, we used world standard population as a reference population.^14^ The age‐specific incidence rates for each province were first determined, and they were then weighted based on the percentage of the world standard population in each age group. The ASIR for each province was then calculated by adding the weighted age‐specific rates. Every rate was given in terms of 100 000 persons per year.

### 2.4 Statistical analysis

The methods and statistical analysis used in this study to calculate cancer incidence, crude rate, and age‐standardized rate were by the cancer project study protocol.^15^, ^16^ We used the direct standardization method to calculate the age‐standardized rate, using world standard population as a reference population. Joinpoint Trend Analysis software 4.9.0.0, developed by IMS, Inc. under contract for the National Cancer Institute, was used to perform joinpoint analysis and calculate the crude and age‐standardized rates for cervical cancer in 2016. In addition, the collected data were analyzed using ArcGIS version 10.5 software (developed by Environmental Systems Research Institute–ESRI). We examined the geographical distribution and clustering of cervical cancer incidence using Moran's index. Moran's index is a spatial statistical instrument employed to evaluate the aggregation and spatial distribution of data within a given geographic area. It helps determine whether similar data points are closer or farther apart than expected in space. If Moran's index is close to positive, it indicates that similar data points are closer to each other than expected and exhibit clustering. Conversely, if it is close to negative, it suggests that data points are typically farther apart from each other than expected.^17^

## 3 RESULTS

We examined 808 registered cases of cervical cancer, and the median age was determined to be 52.19 years (IQR≈1.35). The crude incidence rate of cervical cancer in the entire country was 1.88 per 100 000, while the ASR was 1.90 per 100 000. The highest provincial ASR was observed in Golestan (5.03), East Azerbaijan (4.07), and Ilam (3.72) (Table 1).

**TABLE 1:** Age‐specific incidence (15–49 years/50 and above)(ASIR), crude incidence rate, and ASR of cervical cancer in Iran in 2016 in terms of province of residence.

Province of residence	Number	Crude incidence rate (per 100 000)	Age‐specific incidence rate (ASIR) (per 100 000)		ASR (per 100 000)
			15–49 years old	50 years old and older	
Alborz	17	1.27	1.12	3.19	1.18
Ardabil	15	2.42	2.23	5.95	2.34
Azerbaijan Gharbi	24	1.49	1.1	4.87	1.51
Azarbaijan Shargi	89	4.63	3.88	11.66	4.07
Bushehr	7	1.29	1.28	3.76	1.34
Chaharmahal and Bakhtiari	6	1.29	1.13	3.75	1.39
Esfahan	48	1.90	1.23	5.71	1.70
Fars	24	1.00	0.72	3.09	0.97
Gilan	22	1.74	0.42	5.79	1.35
Golestan	43	4.62	3.04	17.01	5.03
Hamadan	20	2.33	1.84	6.13	2.13
Hormozgan	16	1.84	0.8	10.7	2.60
Ilam	10	3.51	1.73	15.02	3.72
Kerman	40	2.59	2.38	7.69	2.79
Kermanshah	19	1.97	0.71	7.64	2.01
Khorasan North	11	2.56	2.09	7.95	2.39
Khorasan Razavi	83	2.60	2.28	7.59	2.75
Khorasan South	8	2.11	0.98	8.62	2.27
Khuzestan	51	2.20	1.33	9.55	2.62
Kohgiluyeh and Boyer‐Ahmad	3	0.85	0.97	2	1.01
Kordestan	4	0.51	0	2.81	0.48
Lorestan	7	0.81	1.19	0.65	0.70
Markazi	12	1.71	1.49	4.01	1.45
Mazandaran	39	2.39	1.81	5.84	1.90
Qazvin	14	2.25	1.92	6.13	1.98
Qom	10	1.58	1.08	5.94	1.84
Semnan	4	1.16	0.49	4.41	1.11
Sistan and Baluchestan	13	0.95	0.69	5.97	1.64
Tehran	137	2.08	1.49	5.67	1.86
Yazd	10	1.81	2.28	3.16	1.73
Zanjan	2	0.38	0.67	0	0.29

**FIGURE 1:**
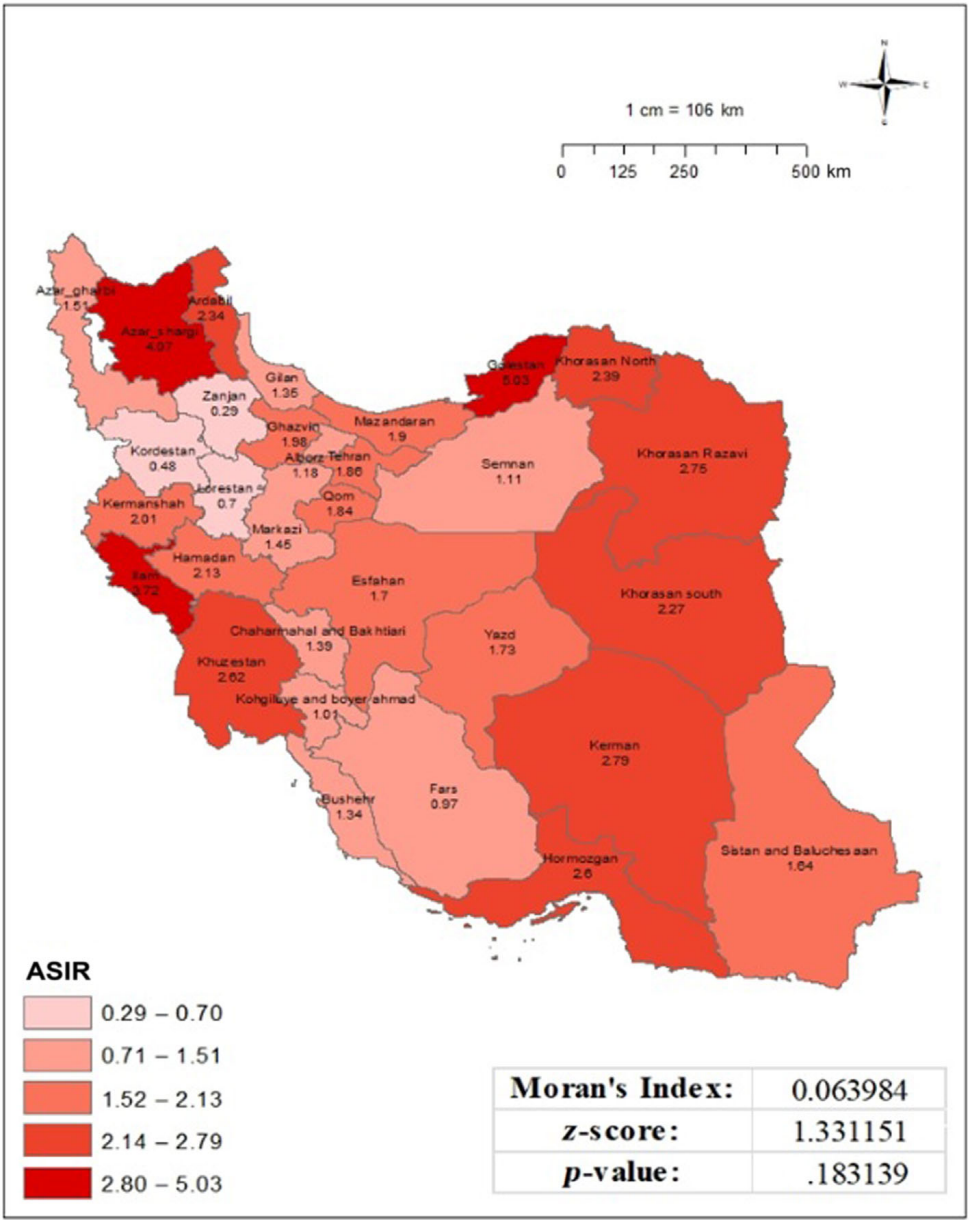
Age‐standardized incidence of cervical cancer per 100 000 populations in Iran in 2016, based on national cancer registry data.

The crude incidence rate of cervical cancer varied from 0.38 to 4.6 per 100 000 persons‐year, with the highest rates observed in East Azerbaijan, Golestan, and Ilam provinces. However, Moran's index did not indicate a significant clustering geographical distribution of crude incidence rate (Moran's index: 0.007, *p*‐value: .57). Meanwhile, the ASR of cervical cancer ranged from 0.29 to 5.03 per 100 000 populations. The calculated Moran's index of 0.064 with a corresponding *p*‐value of .18 indicates no statistically significant spatial clustering or distribution of the analyzed data. In other words, cervical cancer incidence rates do not exhibit a significant geographic pattern or clustering. (Figure 1).

The age‐specific incidence rate of cervical cancer among individuals aged 50 years and older ranged from 0.00 to 17.01 per 100 000 populations, with the highest rates observed in East Azerbaijan, Golestan, and Ilam provinces.

**FIGURE 2:**
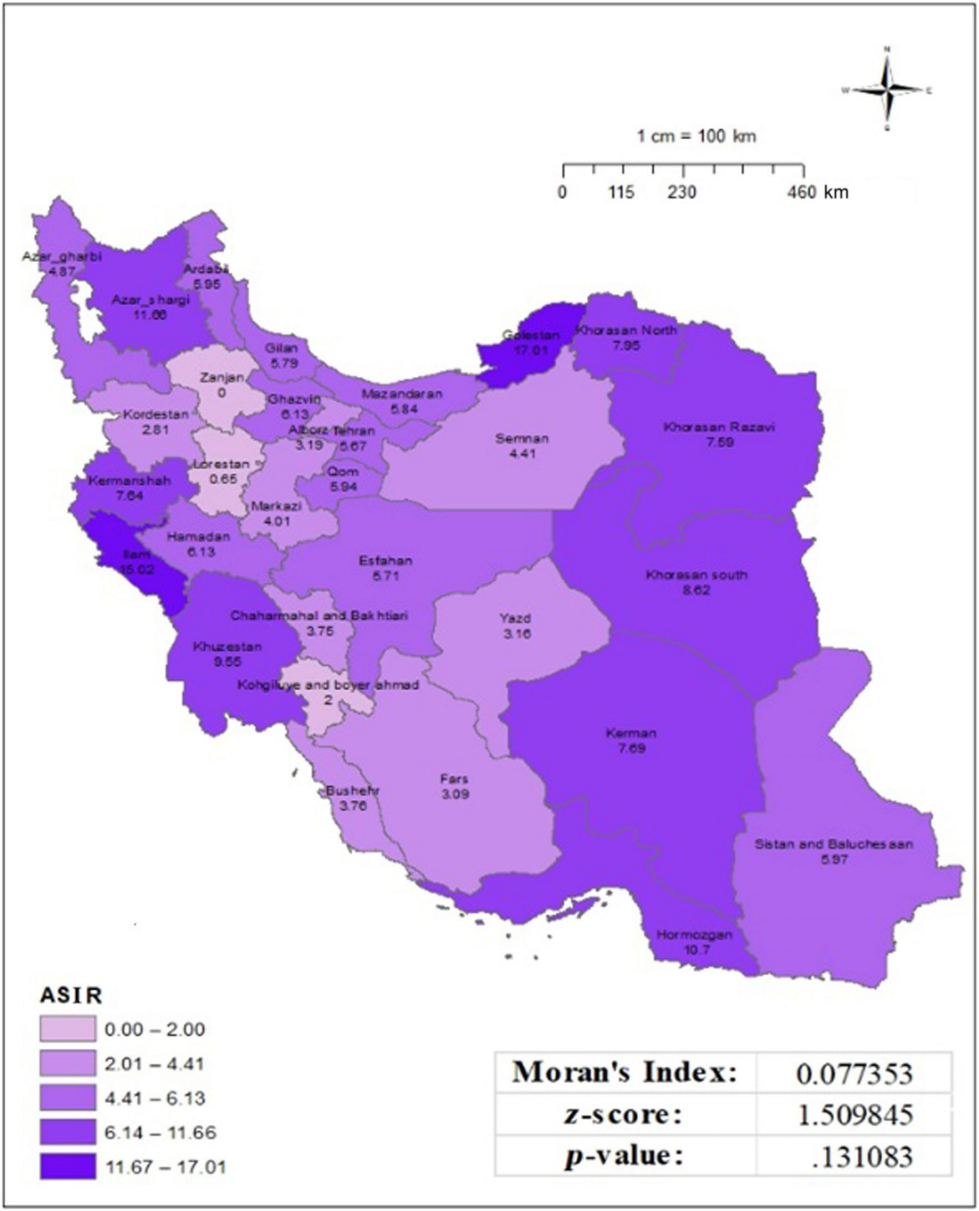
Age‐specific incidence rate (50 years and older) of cervical cancer per 100 000 populations in Iran in 2016, based on national cancer registry data.

**FIGURE 3:**
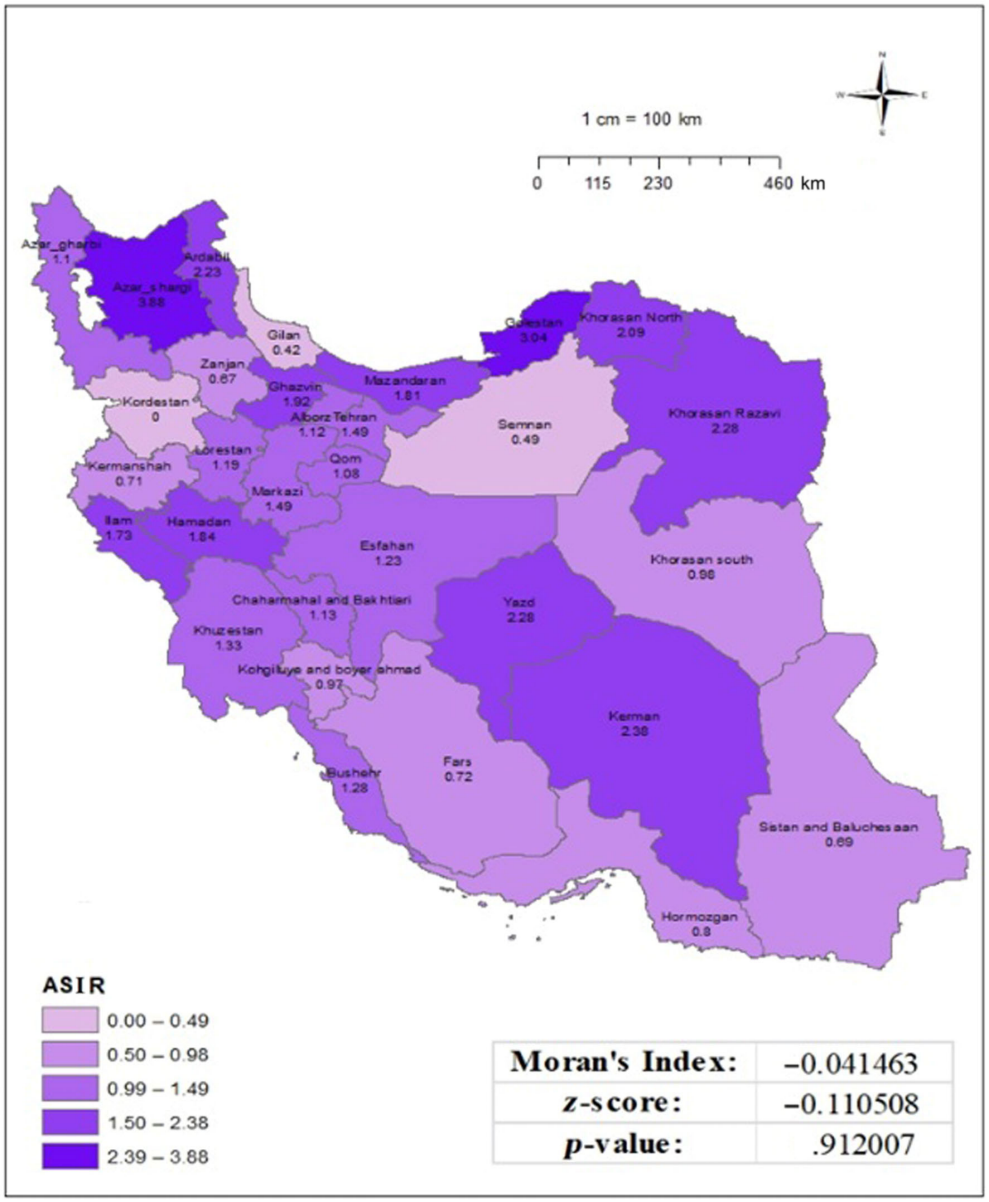
Age‐specific incidence rate (15–49 years) of cervical cancer per 100 000 populations in Iran in 2016, based on national cancer registry data.

However, Moran's index did not indicate a significant clustering geographical distribution of this incidence in the country (Moran's index: 0.077, *p*‐value: .13) (Figure 2). Meanwhile, the age‐specific incidence rate for the individuals aged 15–49 years ranged from 0.00 to 3.88 per 100 000 populations, with the highest rates observed in East Azerbaijan and Golestan provinces. The computed Moran's index of −0.041, together with a corresponding *p*‐value of .91, indicates that there is no statistically significant regional clustering or distribution seen in the investigated incidence data. Put simply, the occurrence rates of the variable being researched do not show any notable geographical pattern or clustering (Figure 3). Among 808 cervical cancer cases, 685 (84.7%) were diagnosed based on pathological reports with morphological verification (MV), 81 cases (10.1%) were clinically identified, and 42 cases (5.2%) were diagnosed using the death certificate‐only method (DCO). Among the cases diagnosed based on pathological reports, 497 cases (61%) were classified as squamous cell carcinoma (SCC), and 103 cases (12.7%) were classified as adenocarcinoma. Further provincial details are provided in Table 2.

**TABLE 2:** Morphology and diagnosis method of cervical cancer in Iran in 2016 in terms of the province of residence.

Province of residence	Morphology (number (percent))			Diagnosis method (number (percent))		
	SCC	Adenocarcinoma	Other‐undetermined	MV	Clinical	DCO
Alborz	11 (64.7%)	1 (5.88%)	5 (29.41%)	15 (88.23%)	2 (11.76)	0 (0%)
Ardabil	11 (73.33%)	2 (13.33%)	2 (13.33%)	15 (100%)	0 (0%)	0 (0%)
Azerbaijan Gharbi	17 (70.83%)	3 (12.5%)	4 (16.66%)	22 (91.66%)	2 (8.3%)	0 (0%)
Azerbaijan Shargi	58 (65.16%)	10 (12.35%)	21 (23.59%)	75 (84.26%)	6 (6.74%)	8 (8.98%)
Bushehr	5 (71.42%)	0 (0%)	2 (28.57%)	7 (100%)	0 (0%)	0 (0%)
Chaharmahal and Bakhtiari	3 (50%)	1 (16.66%)	2 (33.33%)	6 (100%)	0 (0%)	0 (0%)
Esfahan	27 (56.25%)	13 (27.08%)	8 (16.66%)	44 (91.66%)	3 (6.25%)	1 (2.08%)
Fars	12 (50%)	4 (16.66%)	8 (33.33%)	21 (87.5%)	2 (8.33%)	1 (4.16%)
Gilan	12 (54.54%)	0 (0%)	10 (45.45%)	13 (59.09%)	4 (18.18%)	5 (22.72%)
Golestan	29 (67.44%)	3 (6.97%)	11 (25.58%)	35 (81.39%)	7 (16.27%)	1 (2.32%)
Hamadan	12 (60%)	4 (20%)	4 (20%)	18 (90%)	2 (10%)	0 (0%)
Hormozgan	6 (37.5%)	2 (12.5%)	8 (50%)	12 (75%)	4 (25%)	0 (0%)
Ilam	2 (20%)	2 (20%)	6 (60%)	8 (80%)	1 (10%)	1 (10%)
Kerman	29 (72.5%)	3 (7.5%)	8 (20%)	34 (85%)	5 (12.5%)	1 (2.5%)
Kermanshah	14 (73.68%)	0 (0%)	5 (26.31%)	17 (89.47%)	0 (0%)	2 (10.52%)
Khorasan North	2 (18.18%)	2 (18.18%)	7 (63.63%)	6 (54.54%)	4 (36.36%)	1 (9.09%)
Khorasan Razavi	62 (74.69%)	9 (10.84%)	12 (14.45%)	78 (93.97%)	2 (2.4%)	3 (3.61%)
Khorasan South	2 (25%)	1 (12.5%)	5 (62.5%)	4 (50%)	4 (50%)	0 (0%)
Khuzestan	39 (76.47%)	3 (5.88%)	9 (17.64%)	47 (92.15%)	3 (5.88%)	1 (1.96%)
Kohgiluyeh and Boyer‐Ahmad	2 (66.66%)	0 (0%)	1 (33.33%)	3 (100%)	0 (0%)	0 (0%)
Kordestan	4 (100%)	0 (0%)	0 (0%)	4 (100%)	0 (0%)	0 (0%)
Lorestan	2 (28.57%)	3 (42.85%)	2 (28.57%)	6 (85.71%)	1 (14.28%)	0 (0%)
Markazi	4 (33.33%)	1 (8.33%)	7 (58.33%)	8 (66.66%)	0 (0%)	4 (33.33%)
Mazandaran	21 (53.84%)	11 (28.2%)	7 (17.94%)	36 (92.3%)	2 (5.12%)	1 (2.56%)
Qazvin	12 (85.71%)	0 (0%)	2 (14.28%)	12 (85.71%)	0 (0%)	2 (14.28%)
Qom	1 (10%)	4 (40%)	5 (50%)	5 (50%)	3 (30%)	2 (20%)
Semnan	2 (50%)	0 (0%)	2 (50%)	2 (50%)	2 (50%)	0 (0%)
Sistan and Baluchestan	7 (53.84%)	2 (15.38%)	4 (30.76%)	11 (84.61%)	2 (15.38%)	0 (0%)
Tehran	80 (58.39%)	19 (13.86%)	38 (27.73%)	109 (79.56%)	20 (14.59%)	8 (5.83%)
Yazd	7 (70%)	0 (0%)	3 (30%)	10 (100%)	0 (0%)	0 (0%)
Zanjan	2 (100%)	0 (0%)	0 (0%)	2 (100%)	0 (0%)	0 (0%)
Total	497 (61.5%)	103 (12.74%)	208 (25.74%)	81 (10.02%)	685 (84.77%)	42 (5.19%)

Abbreviations: DCO, death certificate‐only; MV, morphological verification; SCC, squamous cell carcinoma.

We did not observe any significant clustering geographical distribution of morphology, diagnostic method, or age groups (Figures S1–S3).

## 4 DISCUSSION

Cervical cancer is an invasive and common cancer with different incidence and prevalence in other regions of the world.^18^, ^19^, ^20^ Arbyn et al. reported worldwide cervical cancer incidence from 4.1 to 43.1 per 100 000 people in 2018.^2^

One study in Jordan announced the average ASR to be 2 per 100 000, with an ASR of 2.1 per 100 000 in 2000 and 1.5 per 100 000 in 2013, with a decrease of 28.6% in the 14 years.^21^ In Iran, cervical cancer ASR ranged from 1.64 to 2.61 per 100 000 during 2003–2009.^7^ Based on the results of this study, ASIR for cervical cancer was 1.9 per 100 000 in 2016. Provincial ASR ranged from 0.29 to 5.03 per 100 000 populations in 2016, and the highest rates were related to Golestan (5.03), East Azerbaijan (4.07), and Ilam (3.72). The above findings showed that cervical cancer ASR during 2003–2009 and in 2016 in Iran has not been high compared with other parts of the world.

The results of previous research indicated that the ASR was 2.5 per 100 000 people in pathology‐based cancer registries compared with 6 out of every 100 000 people in population‐based cancer registries in 2013.^22^ Meanwhile, from 2003 to 2009, while the national cancer registry was mainly pathologic based, the ASR of cervical was incremental which was higher than ASR in 2016. In other words, ASR decreased in 2016 compared with 2003–2009 despite changing the registry system to a population‐based cancer registry in the whole country (ASR was reported to be 1.64, 2.12, and 2.17 per 100 000 in 2003, 2006, and 2009 and 1.9 per 100 000 in 2016). It seems that there is a genuine decrease in the occurrence of cervical cancer in Iran compared with 2003–2009. This might be attributed to the improved adherence to health practices, such as the use of contemporary contraceptive techniques. Besides, referring more people for Pap smear tests and timely treatment of cervical cancer precursor lesions may be related to cervical cancer reduction in recent years.^23^

Based on the results of the Vafaeinezhad et al. study, the highest ASR in 2003 was reported for Tehran (4.24 per 100 000) and Yazd (2.67 per 100 000). In addition, Khorasan Razavi (3.66 per 100 000) and Isfahan (2.84 per 100) in 2006 and Yazd (7.14 per 100 000) in 2009 had the highest ASR in Iran.^5^ Moreover, although the incidence of cervical cancer was high in the central regions of Iran in the past (2000s), the highest statistics belonged to three border provinces of the country in 2016. There are a number of potential causes for the regional differences in cervical cancer incidence. This might include greater rates of identification via Pap smear tests and a greater frequency of risk factors. More research is required to fully examine this issue. Socioeconomic disparities may be another element contributing to regional variances, in addition to risk factors and detection rates. Variances in income, education, and access to healthcare services across different regions can significantly affect the prevalence of risk factors and the ability to access screening and early detection services. Furthermore, differences in healthcare infrastructure, such as the availability of screening facilities, may play a pivotal role in shaping geographical disparities. Cultural and behavioral factors, including variations in the cultural beliefs, lifestyle choices, and healthcare‐seeking behaviors, are additional considerations that can influence the incidence and detection of cervical cancer, and such factors may affect the interpretation of the data.

In the Sharkas et al. study, 46.5% of cervical cancer cases were SCC type.^21^ In Mortazavi et al. study, 87% of cervical cancer cases were SCC (87 out of 100 cervical cancer cases).^24^ In Sadeghi et al.'s study, SCC accounted for 73% of patients and adenocarcinoma in 7.6%, poorly differentiated carcinoma in 11.5%, and situ carcinoma (CINIII) in 7.6% of cervical cancer cases. ^25^ SCC was the most common type of cervical cancer (61%), consistent with the results of other studies.

It is important to note that, apart from SCC, adenocarcinoma of the cervix accounts for approximately 20%–25% of all cervical malignancies. Notably, SCC has a higher frequency of PD‐L1 expression compared with adenocarcinoma. Moreover, it is important to note that in patients with SCC, widespread PD‐L1 expression is correlated with unfavorable disease‐free survival and disease‐specific survival, in contrast to limited PD‐L1 expression, which is associated with a much better prognosis. On the other hand, in adenocarcinoma, there appears to be a survival benefit for patients with tumors lacking PD‐L1‐positive tumor‐associated macrophages.^26^

The results of this study showed that the ASIR of cervical cancer among people aged 50 years and older ranged from 0.00 to 17.01 per 100 000 populations, with the highest rates observed in East Azerbaijan, Golestan, and Ilam provinces. This can be attributed to several factors. Cervical cancer increases with age in terms of the cumulative exposure to risk factors, such as persistent infection with high‐risk types of human papillomavirus (HPV). Older individuals may not have had access to cervical cancer screening, like Pap smears, in their earlier years and might not have benefited from more recent HPV vaccinations. Furthermore, the slower progression of cervical cancer can result in delayed diagnoses in older individuals, as symptoms may not become evident until the disease has advanced. Older generations may have less awareness of the importance of regular screenings, HPV vaccinations, and the risk factors associated with cervical cancer. Disparities in healthcare access may have an impact on older people's timely cervical cancer detection and treatment. Healthcare services may be harder to get in certain areas, which might lead to insufficient or delayed treatment. Lifestyle factors, such as smoking, poor nutrition, and a lack of exercise, may have been more prevalent in certain regions, increasing the risk of cervical cancer in older individuals.^27^, ^28^ Furthermore, the genetic and environmental factors specific to these provinces could increase the risk of cervical cancer in older age groups. However, further research is necessary to identify these factors.

The quality of cancer registry system for cervical cancer was relatively high (MV:84.7 and DCO:5.1) compared with all cancer site registration quality in Iran and some other parts of the world.^29^ Besides, provincial MV and DCO were acceptable. Therefore, it can be assumed that data on cervical cancer and derived indices in 2016 in Iran were reliable.

Several limitations associated with this study should be considered when interpreting the results. First, the study only examined registered cases of cervical cancer, which may not provide a complete picture of the true incidence of disease in terms of the potential underreporting or misclassification. This might cause the real incidence rates to be overestimated or underestimated. Second, data on possible risk factors for cervical cancer, such as smoking, sexual activity, and HPV infection, were not included in the research. Including this information could have provided important insights into the observed incidence rates and potential prevention strategies. These days, there is increasing attention to immunotherapy, such as adoptive T‐cell therapy, and vaccine‐based therapies in cervical cancer treatment, as the causative role of HPV infection in cervical cancer initiation and progression is well understood.^30^

Thirdly, the study was conducted within a specific period (2016), which may not reflect current trends in cervical cancer incidence or mortality. The results should be cautiously interpreted when considering more recent data. These limitations suggest that further research is needed to understand cervical cancer's burden better and identify potential risk factors and prevention strategies.

## 5 CONCLUSION

The incidence of cervical cancer in Iran was lower than the global average, and we did not identify any significant disparities in the incidence rates among the provinces. The study revealed that most cervical cancer cases were diagnosed based on pathological reports with the MV, with SCC being the most common type.

Furthermore, based on these results, in provinces with higher incidence rates, implementing more intensive screening programs emphasizing early detection and prevention can be a valuable step. Additionally, implementing educational programs and public health campaigns may aid in increasing knowledge of the significance of routine screenings as well as the risk factors for cervical cancer. Reducing gaps in early detection requires ensuring fair access to healthcare services, including Pap smear testing, in every province. Promoting HPV vaccination, especially in regions with higher incidence rates, is crucial as HPV is a significant risk factor for cervical cancer.

## AUTHOR CONTRIBUTIONS

**Alireza Anjam Majoumerd:** Conceptualization (equal); data curation (equal); investigation (equal); methodology (equal); resources (equal); software (equal); supervision (equal). **Saeed Hosseini:** Conceptualization (equal); formal analysis (equal); methodology (equal); resources (equal); software (equal); validation (equal); writing – original draft (equal). **Seyyed Mohammad Hossein Hosseini:** Conceptualization (equal); data curation (equal); formal analysis (equal); methodology (equal); software (equal); validation (equal); writing – original draft (equal). **Gholamreza Roshandel:** Conceptualization (equal); data curation (equal); formal analysis (equal); investigation (equal); methodology (equal); software (equal); validation (equal). **Vahid Rahmanian:** Formal analysis (equal); investigation (equal); methodology (equal); software (equal); validation (equal); visualization (equal); writing – original draft (equal); writing – review and editing (equal). **Narjes Hazar:** Conceptualization (equal); formal analysis (equal); methodology (equal); project administration (equal); resources (equal); software (equal); supervision (equal); validation (equal); visualization (equal); writing – original draft (equal); writing – review and editing (equal).

## CONFLICT OF INTEREST STATEMENT

The authors have stated explicitly that there are no conflicts of interest in connection with this article.

## ETHICS STATEMENT

It states that the study was presented to the Ethics Committee of Shahid Sadoughi University of Medical Sciences and received approval under the number IR.SSU.MEDICINE.REC.1399.115. Besides, we adhered to the principles of the Helsinki Declaration throughout the study. To protect the privacy of individuals, we made sure to conceal their names and surnames. The data used in the study were extracted from the cancer registry system using unique identification codes.

## Supporting information


**Figure S1.** Geographical distribution status of the frequency of cervical cancer age groups in Iran in 2016.
**Figure S2.** Geographical distribution status of cervical cancer morphology frequency in Iran in 2016.
**Figure S3.** Geographical distribution status of cervical cancer diagnosis in Iran in 2016.

## Data Availability

Upon a reasonable request, the corresponding author can provide access to the data supporting this study's findings. The data used in this research are available upon request. Researchers interested in accessing the data may contact the corresponding author via email at (narjeshazar@yahoo.com). We are committed to facilitating access to our data for academic and research purposes while ensuring compliance with ethical and legal guidelines governing data sharing.
