# Supplementary material for: Epidemiology of cervical cancer in Iran in 2016: A nationwide study of incidence and regional variation
Source: Cancer Rep (Hoboken). 2024 Jan 12;7(2):e1973. doi: 10.1002/cnr2.1973 (PMC10849997; doi:10.1002/cnr2.1973)
Supplement: Supplementary file 1 — Figure S1. Geographical distribution status of the frequency of cervical cancer age groups in Iran in 2016. Figure S2. Geographical distribution status of cervical cancer morphology frequency in Iran in 2016. Figure S3. Geographical distribution status of cervical cancer diagnosis in Iran in 2016. [file CNR2-7-e1973-s001.docx]

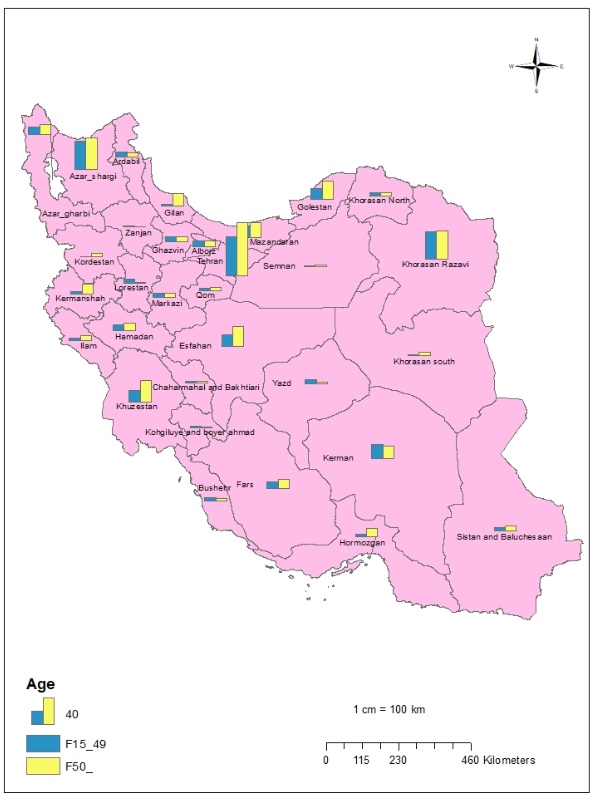


Suppl. Figure 1- Geographical distribution status of the frequency of cervical cancer age groups in Iran in 2016


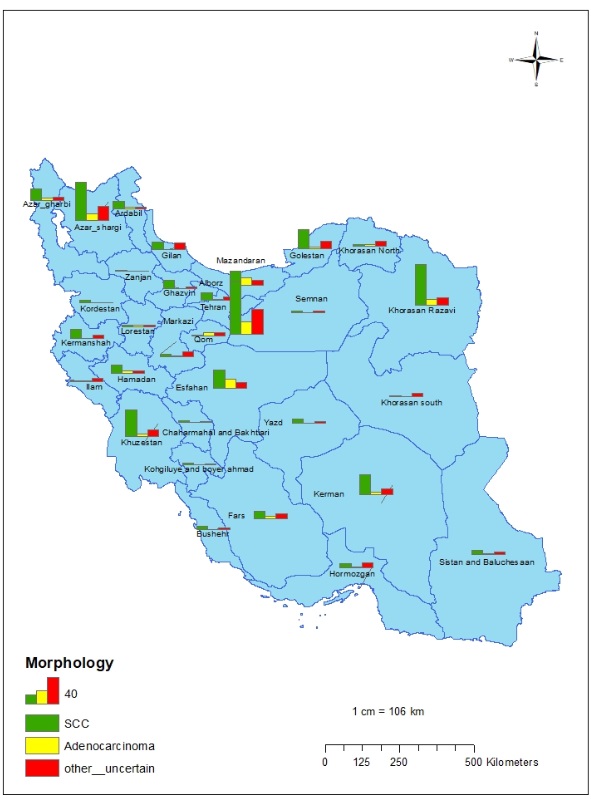


Suppl. Figure 2- Geographical distribution status of cervical cancer morphology frequency in Iran in 2016


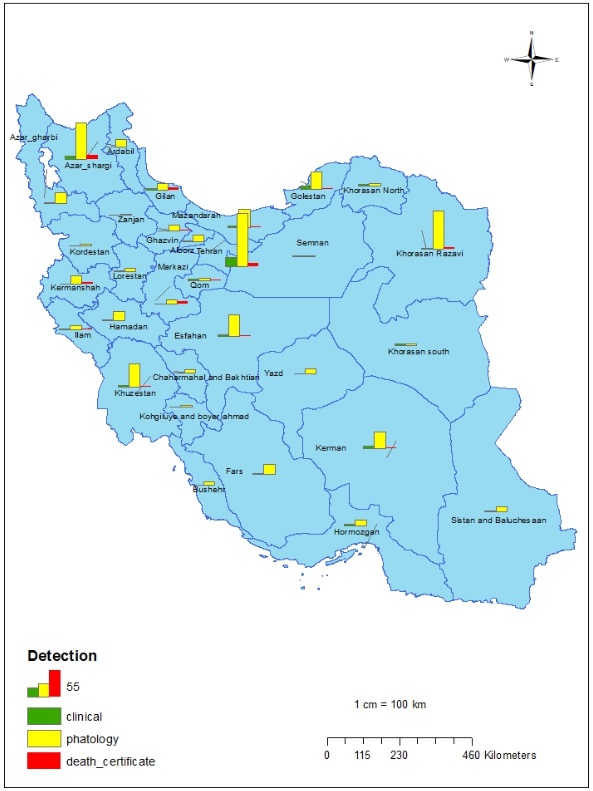


Suppl. Figure 3- Geographical distribution status of cervical cancer diagnosis in Iran in 2016
